# Supplementary material for: Effects of Host Plants and Their Infection Status on Acquisition and Inoculation of A Plant Virus by Its Hemipteran Vector
Source: Pathogens. 2023 Sep 1;12(9):1119. doi: 10.3390/pathogens12091119 (PMC10537197; doi:10.3390/pathogens12091119)
Supplement: Supplementary file 1 [file pathogens-12-01119-s001.zip › pathogens-2543524-supplementary.pdf]

pathogens

MDPI

S.1 Clip Cage

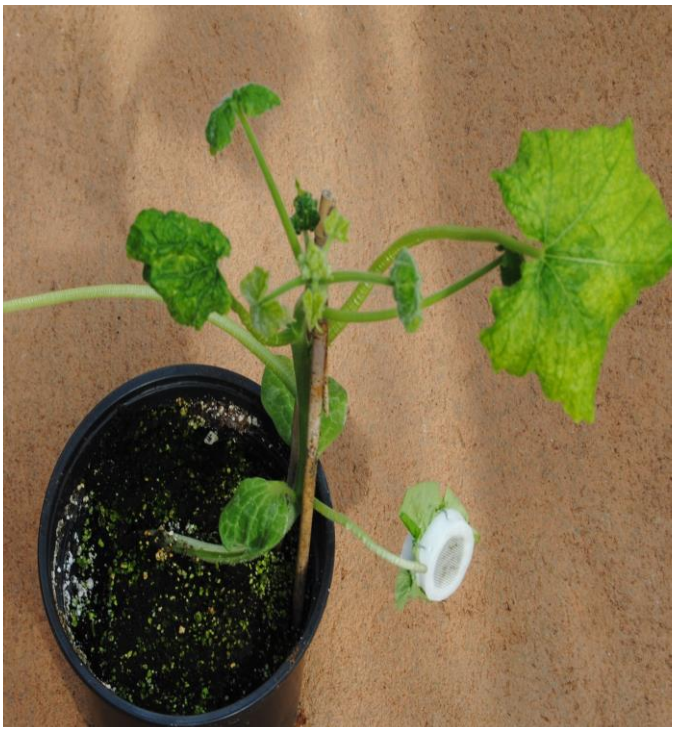

S.2 Virus accumulation in plants (E1).

Exp Repeat	Replication	Plant	Copies per ng DNA
1	1	Snap bean	4.09E+04
1	2	Snap bean	2.73E+04
1	3	Snap bean	6.26E+04
1	4	Snap bean	4.12E+05
1	5	Snap bean	2.76E+05
1	6	Snap bean	6.07E+04
2	1	Snap bean	1.99E+05
2	2	Snap bean	4.10E+05
2	3	Snap bean	3.01E+04
2	4	Snap bean	1.02E+05
2	5	Snap bean	1.76E+05
2	6	Snap bean	6.00E+04
3	7	Snap bean	8.40E+04
3	1	Snap bean	2.31E+05
3	2	Snap bean	3.22E+05
3	3	Snap bean	4.30E+05
3	4	Snap bean	2.03E+05
3	5	Snap bean	2.26E+05
3	6	Snap bean	5.12E+05

1	1	Tobacco	2.15E+06
1	2	Tobacco	9.33E+06
1	3	Tobacco	9.27E+06
1	4	Tobacco	9.53E+06
1	5	Tobacco	8.40E+05
1	6	Tobacco	3.17E+06
1	7	Tobacco	2.12E+06
2	1	Tobacco	2.72E+06
2	2	Tobacco	3.29E+06
2	3	Tobacco	1.53E+06
2	4	Tobacco	3.34E+06
2	5	Tobacco	7.55E+05
2	6	Tobacco	9.72E+05
2	7	Tobacco	8.60E+06
3	1	Tobacco	7.10E+06
3	2	Tobacco	5.27E+06
3	3	Tobacco	7.40E+07
3	4	Tobacco	5.82E+06
3	5	Tobacco	2.11E+06
3	6	Tobacco	1.36E+06
3	7	Tobacco	8.40E+06
3	8	Tobacco	9.04E+06
3	9	Tobacco	6.41E+06
1	1	Squash	4.89E+10
1	2	Squash	5.51E+10
1	3	Squash	1.65E+09
1	4	Squash	4.48E+09
1	5	Squash	5.55E+09
1	6	Squash	1.56E+10
1	7	Squash	3.88E+09
1	8	Squash	3.73E+09
2	1	Squash	1.29E+10
2	2	Squash	1.14E+10
2	3	Squash	5.26E+10
2	4	Squash	2.31E+09
2	5	Squash	1.29E+09
2	6	Squash	1.17E+08
3	7	Squash	3.19E+09
3	1	Squash	1.99E+07
3	2	Squash	5.25E+10
3	3	Squash	8.93E+09

3	4	Squash	1.48E+08
3	5	Squash	8.13E+09
3	6	Squash	2.30E+09
3	7	Squash	2.96E+09

S.3 Virus accumulation in plants (E2).

Exp Repeat	Replication	Plant	Copies per ng DNA
1	1	Squash from Bean	7.27E+05
1	2	Squash from Bean	7.57E+05
1	3	Squash from Bean	1.42E+04
1	4	Squash from Bean	1.62E+05
1	5	Squash from Bean	4.49E+03
1	6	Squash from Bean	1.99E+03
1	7	Squash from Bean	2.76E+03
2	1	Squash from Bean	1.54E+03
2	2	Squash from Bean	7.15E+03
2	3	Squash from Bean	6.22E+05
2	4	Squash from Bean	5.55E+05
2	5	Squash from Bean	4.42E+04
2	6	Squash from Bean	2.02E+05
2	7	Squash from Bean	2.49E+03
2	8	Squash from Bean	2.09E+03
2	9	Squash from Bean	2.26E+03
2	10	Squash from Bean	5.14E+03
3	1	Squash from Bean	5.55E+03
3	2	Squash from Bean	5.57E+05
3	3	Squash from Bean	1.42E+04
3	4	Squash from Bean	9.90E+04
3	5	Squash from Bean	4.29E+03
3	6	Squash from Bean	3.99E+03
3	7	Squash from Bean	1.02E+05
3	8	Squash from Bean	1.64E+03
3	9	Squash from Bean	7.74E+03
1	1	Squash from Tobacco	8.90E+10
1	2	Squash from Tobacco	8.04E+10
1	3	Squash from Tobacco	1.25E+11
1	4	Squash from Tobacco	9.56E+10
1	5	Squash from Tobacco	9.35E+08
1	6	Squash from Tobacco	1.11E+11
2	1	Squash from Tobacco	1.68E+11
2	2	Squash from Tobacco	1.30E+11

2	3	Squash from Tobacco	1.57E+11
2	4	Squash from Tobacco	2.20E+10
2	5	Squash from Tobacco	6.04E+10
2	6	Squash from Tobacco	2.25E+11
2	7	Squash from Tobacco	8.66E+10
2	8	Squash from Tobacco	2.56E+09
2	9	Squash from Tobacco	1.01E+11
2	10	Squash from Tobacco	1.48E+11
3	1	Squash from Tobacco	2.00E+11
3	2	Squash from Tobacco	2.77E+11
3	3	Squash from Tobacco	1.30E+11
3	4	Squash from Tobacco	1.57E+11
3	5	Squash from Tobacco	4.12E+10
3	6	Squash from Tobacco	3.04E+10
3	7	Squash from Tobacco	1.06E+12
1	1	Squash from squash	5.37E+11
1	1	Squash from squash	8.13E+10
1	2	Squash from squash	8.43E+10
1	3	Squash from squash	1.83E+11
1	4	Squash from squash	2.93E+11
1	5	Squash from squash	2.50E+10
1	6	Squash from squash	5.60E+10
1	7	Squash from squash	5.82E+10
2	1	Squash from squash	2.50E+10
2	2	Squash from squash	1.37E+12
2	3	Squash from squash	6.13E+10
2	4	Squash from squash	2.22E+11
2	5	Squash from squash	5.37E+11
2	6	Squash from squash	8.13E+10
2	7	Squash from squash	8.43E+10
2	8	Squash from squash	1.83E+11
2	9	Squash from squash	2.93E+11
3	1	Squash from squash	1.00E+10
3	2	Squash from squash	4.20E+10
3	3	Squash from squash	4.92E+10
3	4	Squash from squash	5.37E+11
3	5	Squash from squash	8.13E+10
3	6	Squash from squash	8.43E+10
3	7	Squash from squash	1.83E+11
3	8	Squash from squash	2.93E+11
3	9	Squash from squash	2.50E+10

3	10	Squash from squash	5.60E+10

S.4 Virus accumulation in plants (E3).

Exp Repeat	Replica-tion	Plant*	Copies per ng DNA
1	1	SBS	26510
1	2	SBS	56000
1	3	SBS	14100
2	1	SBS	42000
2	2	SBS	42940
3	1	SBS	19920
3	2	SBS	14200
3	3	SBS	62000
3	4	SBS	14200
1	1	STS	8.90E+10
1	2	STS	8.04E+10
1	3	STS	1.25E+11
1	4	STS	9.56E+10
1	5	STS	9.35E+11
1	6	STS	8.11E+10
1	7	STS	1.68E+10
1	8	STS	1.30E+10
2	1	STS	5.57E+10
2	2	STS	2.32E+10
2	3	STS	4.32E+10
2	4	STS	5.67E+11
2	5	STS	8.99E+11
2	6	STS	5.67E+10
2	7	STS	9.56E+10
2	8	STS	7.56E+10
2	9	STS	2.03E+11
3	1	STS	4.56E+11
3	2	STS	1.30E+11
3	3	STS	5.70E+11
3	4	STS	5.57E+10
3	5	STS	2.32E+10
3	6	STS	4.32E+10
3	7	STS	5.67E+11
3	8	STS	8.99E+11
1	1	SMS	5.37E+11

1	2	SMS	8.13E+10
1	3	SMS	8.43E+10
1	4	SMS	3.83E+11
1	5	SMS	2.93E+11
1	6	SMS	2.50E+11
1	7	SMS	5.60E+10
1	8	SMS	5.82E+10
1	9	SMS	2.50E+10
1	10	SMS	8.90E+10
2	1	SMS	8.04E+10
2	2	SMS	2.51E+11
2	3	SMS	7.66E+10
2	4	SMS	7.35E+10
2	5	SMS	6.54E+11
2	6	SMS	3.68E+11
3	1	SMS	2.60E+11
3	2	SMS	6.34E+11
3	3	SMS	4.11E+11
3	4	SMS	3.24E+10
3	5	SMS	5.60E+10
3	6	SMS	5.82E+10
3	7	SMS	2.50E+10
3	8	SMS	8.90E+10

*

SBS squash infected from squash that was originally infected from snap bean. STS squash infected from squash that was originally infected from tobacco.

SMS squash infected from squash that was originally infected from mixed infected squash.
